# How, When, and Where Relic DNA Affects Microbial Diversity

**DOI:** 10.1128/mBio.00637-18

**Published:** 2018-06-19

**Authors:** J. T. Lennon, M. E. Muscarella, S. A. Placella, B. K. Lehmkuhl

**Affiliations:** a Department of Biology, Indiana University, Bloomington, Indiana, USA; b W. K. Kellogg Biological Station, Michigan State University, East Lansing, Michigan, USA; University of Oklahoma

**Keywords:** biodiversity, ecology, extracellular DNA, mathematical modeling, phylogenetic analysis, sampling theory

## Abstract

Extracellular or “relic” DNA is one of the largest pools of nucleic acids in the biosphere. Relic DNA can influence a number of important ecological and evolutionary processes, but it may also affect estimates of microbial abundance and diversity, which has implications for understanding environmental, engineered, and host-associated ecosystems. We developed models capturing the fundamental processes that regulate the size and composition of the relic DNA pools to identify scenarios leading to biased estimates of biodiversity. Our models predict that bias increases with relic DNA pool size, but only when the species abundance distributions (SADs) of relic and intact DNA are distinct from one another. We evaluated our model predictions by quantifying relic DNA and assessing its contribution to bacterial diversity using 16S rRNA gene sequences collected from different ecosystem types, including soil, sediment, water, and the mammalian gut. On average, relic DNA made up 33% of the total bacterial DNA pool but exceeded 80% in some samples. Despite its abundance, relic DNA had a minimal effect on estimates of taxonomic and phylogenetic diversity, even in ecosystems where processes such as the physical protection of relic DNA are common and predicted by our models to generate bias. Our findings are consistent with the expectation that relic DNA from different taxa degrades at a constant and equal rate, suggesting that it may not fundamentally alter estimates of microbial diversity.

## IMPORTANCE

The ability to rapidly obtain millions of gene sequences and transcripts from a range of environments has greatly advanced understanding of the processes that regulate microbial communities. However, nucleic acids extracted from complex samples do not come only from viable microorganisms. Dead microorganisms can generate large pools of relic DNA that distort insight into the ecology and evolution of microbial systems. Here, we develop a conceptual and quantitative framework for understanding how relic DNA influences the structure of microbiomes. Our theoretical models and empirical results demonstrate that a large relic DNA pool does not automatically lead to biased estimates of microbial diversity. Rather, relic DNA effects emerge in combination with microscale processes that alter the commonness and rarity of sequences found in heterogeneous DNA pools.

## INTRODUCTION

When microorganisms die, their DNA leaks into the surrounding environment. The fate of this relic DNA has important implications for evolutionary and ecological processes. For example, relic DNA can be taken up and incorporated into the genomes of some microorganisms via transformation, thereby serving as a reservoir of genetic information that can confer new traits and fitness benefits to distantly related taxa (1). In addition, relic DNA is a high-quality resource containing carbon, nitrogen, and phosphorus that is consumed by a diverse array of bacteria with consequences for microbial community structure and ecosystem processes (2, 3).

Relic DNA also has the potential to alter cultivation-independent estimates of diversity, which are widely used for addressing questions concerning the assembly, biogeography, and functioning of microbial communities. Microbial DNA extracted from environmental and host-associated samples is not derived solely from metabolically active organisms (4). A large portion of the individuals in a microbial community is dormant or dead (5, 6). Although nucleic acids can be temporarily retained in nonviable cells, DNA is ultimately released into the environment when individuals die from autolysis, senescence, viral infection, or predation (7). Together, these sources of mortality can create large pools of relic DNA (8, 9). For example, there is an estimated 0.45 Pg of relic DNA in global ocean sediments, which is 70-fold greater than the amount of DNA contained in intact cells from the same environments (2). If included in estimates of biodiversity, relic DNA could distort our understanding of the ecological and evolutionary processes that regulate the distribution, abundance, and function of microbial taxa.

The processes that regulate relic DNA dynamics vary among ecosystems. In well-mixed microbial habitats, the residence time of relic DNA can be short owing to high rates of hydrolysis, oxidation, and UV-mediated damage (9). For example, in surface waters of freshwater and marine environments, the extracellular DNA pool turns over in less than 1 day (10, 11) while plasmid DNA begins to degrade in just minutes (12). As a result, the size distribution of relic DNA is skewed toward small fragments ranging between 100 and 500 bp (13, 14). In more-structured microbial habitats, other factors contribute to the size and turnover of the relic DNA pool. For example, binding with inorganic and organic substances in soils and sediments reduces the rate of relic DNA degradation (8). Likewise, biofilms, aggregates, and outer membrane vesicles can protect relic DNA from hydrolytic enzymes (7, 15, 16). Similarly, the heterogeneous distribution of microorganisms in structured habitats can create “hot spots” of metabolic activity (17) that increase the microscale turnover of relic DNA. Collectively, these processes may help explain the abundance and diversity of relic DNA sequences in a range of ecosystems, including ocean sediments (2), the built environment (18), and the mammalian gastrointestinal tract (19).

To date, the documented effects of relic DNA on estimates of diversity are idiosyncratic. Even in samples with large amounts of relic DNA, bias can be nonexistent or substantial and can lead to the overestimation or underestimation of diversity (20, 21). Such observations reflect the need for a more mechanistic understanding of relic DNA dynamics so that microbial communities can be consistently and accurately characterized across ecosystems. Here, we develop a theoretical framework that considers the processes regulating the size and turnover of the relic DNA pool. We emphasize that the direction and magnitude of bias are influenced by sampling from a joint species abundance distribution (SAD) consisting of sequences from the relic and intact DNA pools. We evaluated our models by quantifying the contribution of relic DNA to the abundance and diversity of bacterial communities in a set of ecosystem types that have contrasting relic DNA dynamics.

## RESULTS

### Theoretical framework for relic DNA dynamics and diversity.

**FIG 1:**
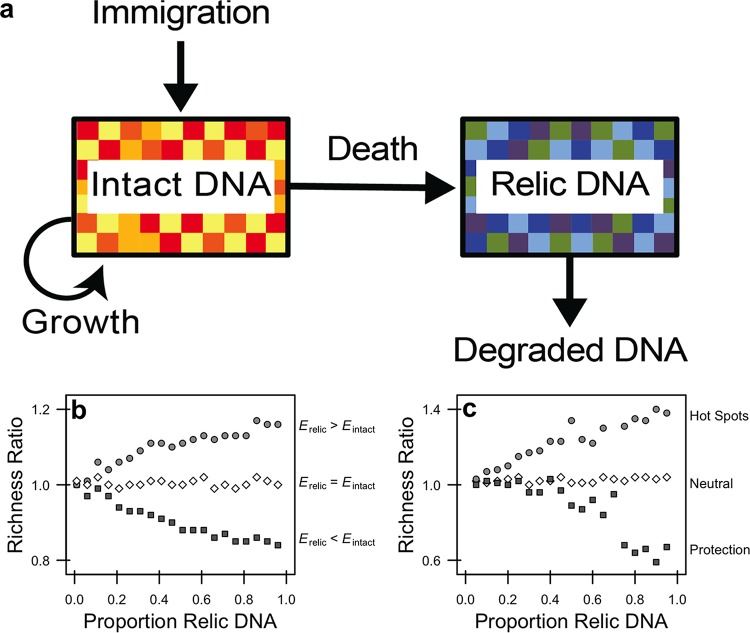
Modeling relic DNA dynamics. (a) The amount of relic DNA in a microbial environment is determined by inputs associated with the mortality of viable individuals with intact DNA and by losses associated with the degradation of relic DNA. If the diversity of sequences contained in the relic DNA pool is sufficiently different from that in the intact DNA pool, then relic DNA may bias estimates of microbial biodiversity (as indicated by different colored boxes) when sampling from the total (intact + relic) DNA pool. (b) We developed a sampling-based simulation model to explore the effects of mixing intact and relic DNA on estimates of diversity. We populated intact and relic communities with individuals from a lognormal species abundance distribution (SAD). We altered the diversity (i.e., evenness, *E*) of the relic community by changing the scale parameter of the lognormal distribution describing the SAD. We then sampled and mixed the intact and relic communities so that the relic contribution to total community ranged from 0.01 to 0.96. (c) To gain mechanistic insight into how bias arises, we created a stochastic process-based model that captures features that influence relic DNA dynamics, including immigration, birth, death, and degradation (a). We simulated a range of degradation rates to achieve relic DNA pool sizes with proportions ranging between 0.05 and 0.95. To explore how degradation alters the SAD of the relic community, we explored three scenarios. First, we simulated a neutral scenario where relic DNA sequences produced by different species degrade at the same rate. Second, we simulated conditions under which physical, chemical, or biological processes reduce the degradation rate of relic DNA belonging to some species via protection. Third, we simulated “hot spots” where more abundant relic DNA sequences experience higher rates of relic DNA degradation, a condition that may arise in structured habitats where there are patchy distributions of individuals and their metabolic products (i.e., enzymes). We ran simulations for 10,000 time steps and then sampled the intact and relic communities. To quantify bias in diversity (b and c), we calculated “richness ratios” which reflect the number of species in the total DNA pool (intact + relic) divided by the number of species in the intact DNA pool in a simulation. When values for richness ratios equal 1, relic DNA has no effect on estimates of diversity; when richness ratios are >1, relic DNA overestimates true diversity; and when richness ratios are <1, relic DNA underestimates true diversity.

We developed a set of interrelated models to identify conditions that we hypothesized would affect relic DNA dynamics and lead to biased estimates of microbial diversity. We began with a conceptual model representing the dynamics of intact and relic DNA (Fig. 1a). The size and composition of the intact DNA pool reflect sequences contained in viable bacteria belonging to different species, which are influenced by births, deaths, and immigration. For the relic DNA pool, the size and composition are determined by the input of sequences associated with the death of bacteria from the intact DNA pool and losses associated with the degradation of relic DNA sequences. With this framework established, we then developed a sampling model to explore how diversity estimates are affected when draws come from a joint distribution of sequences derived from the intact and relic pools. Finally, we integrated our sampling-based model with a process-based model to describe relic DNA dynamics and their effects on diversity estimation.

### (i) Sample-based model.

Simulations from our sampling-based model indicate that a sufficiently large relic DNA pool is required but not sufficient to create bias in estimates of species richness, the number of taxa in a sample (Fig. 1b). Critically, the simulations reveal that there must also be differences between the species abundance distributions (SADs) of the intact and relic DNA pools for bias to arise. When the relic DNA pool has a more even SAD, sampling from the total pool (intact + relic) leads to overestimation of true richness (Fig. 1b). Conversely, when the relic DNA pool has a less even SAD, sampling from the total pool leads to underestimation of true richness (Fig. 1b).

### (ii) Process-based model.

Simulations from our process-based model identify conditions that lead to relic DNA bias. We determined that relic DNA pool size reaches a stable equilibrium when *R* = *m* · *I*/*d*, where *R* is the size (number of sequences) of the relic DNA pool, *m* is the per capita mortality rate of bacteria in the intact DNA pool, *I* is the size (number of sequences) of the intact DNA pool, and *d* is the per capita degradation rate of the relic DNA sequences. From this, it can be shown that *R* increases with the residence time (τ = *R*/*d*) of the relic DNA pool (see Fig. S1 in the supplemental material). When we considered the neutral scenario where the rates at which relic DNA sequences degrade are equivalent among species, we found that the shapes of relic SAD and intact SAD are nearly identical. No bias arises under these conditions, regardless of the relic DNA pool size (Fig. 1c). Next, we simulated physical protection by reducing the degradation rates of relic DNA sequences belonging to randomly selected species (see Materials and Methods). This created a less even relic SAD, resulting in the underestimation of richness in the total DNA pool (Fig. 1c). Finally, we considered the scenario where there are “hot spots” of relic DNA degradation. When simulating accelerated degradation rates for abundant relic DNA sequences, there was a more even relic SAD that resulted in the overestimation of richness in the total DNA pool (Fig. 1c).

10.1128/mBio.00637-18.1 FIG S1  Additional detail on sampling-based and process-based models. Download FIG S1, PDF file, 0.5 MB. Copyright © 2018 Lennon et al. 2018 Lennon et al. This content is distributed under the terms of the Creative Commons Attribution 4.0 International license.

### Empirical assessment of relic DNA abundance and effects on diversity estimation.

Guided by the insight from our model, we obtained samples from replicate sites in four different ecosystem types (soil, sediment, water, and the mammalian gut). Each sample was treated with DNase I in a paired fashion to quantify the abundance of relic DNA and its effect on taxonomic and phylogenetic measures of diversity. We tested the hypothesis that the importance of relic DNA would vary among ecosystems owing to features that might retard or enhance the species-specific degradation of relic DNA sequences. We also tested the prediction that bias should increase with increasing relic DNA pool size.

### (i) Variation in relic DNA pool size.

**FIG 2:**
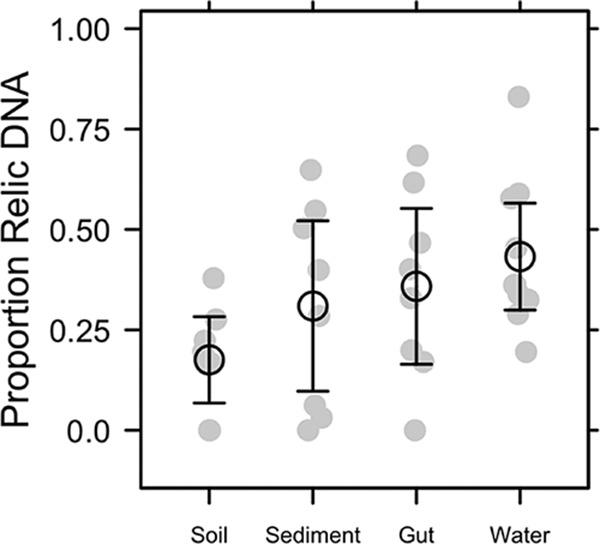
Proportion of bacterial relic DNA in different ecosystem types. We quantified the amount of intact DNA in a sample after removing relic DNA with a DNase treatment. We then estimated the proportion of relic DNA as 1 − (intact DNA/total DNA), where the total DNA concentration was quantified without DNase treatment. Relic DNA constituted an appreciable portion of the total DNA pool but was not affected by the ecosystem type from which the sample was collected (gut, soil, sediment, and water). Gray symbols are the observed data, and black symbols represent means ± 95% confidence intervals.

Relic DNA accounted for a substantial but variable portion of the total bacterial DNA pool. The proportion of relic DNA estimated using quantitative PCR (qPCR) was normally distributed (mean ± standard deviation [SD], 0.33 ± 0.218) and ranged from 0 to 0.83 across 34 samples obtained from different ecosystem types (Fig. 2). Even though host and environmental features associated with different habitats are thought to influence relic DNA dynamics (9, 20), there was only a marginally significant effect of ecosystem type on the proportion of relic DNA (one-way analysis of variance [ANOVA], *F*_3, 30_ = 2.43, *P* = 0.084), which likely reflected the contrast between soil and water samples (Tukey’s honestly significant difference [HSD], adjusted *P* = 0.058). Nevertheless, the large average pool size and range in relic DNA across samples provided us with the opportunity to explore features of our model, specifically the magnitude and direction of bias that relic DNA should have on estimates of microbial diversity.

### (ii) Magnitude and direction of bias on microbial diversity.

**FIG 3:**
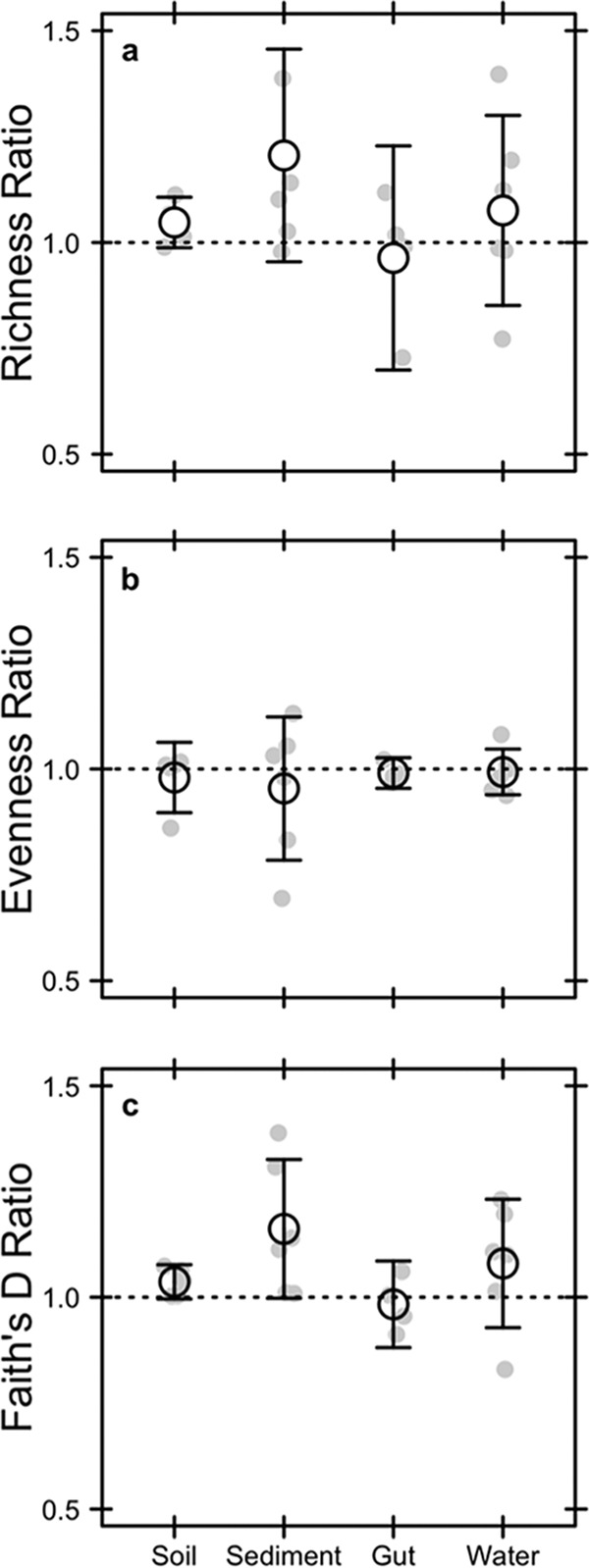
Effect of relic DNA on within-sample (alpha) bacterial diversity in different ecosystem types. We tested for the effects of bias caused by relic DNA by calculating diversity ratios for richness (a), evenness (b), and phylogenetic diversity (c). The ratios reflect the diversity of the total DNA pool (intact + relic) divided by the diversity of the intact DNA pool. Relic DNA did not bias any measures of diversity in any of the ecosystem types. Richness was calculated as the number of operational taxonomic units (97% sequence similarity of the 16S rRNA gene), evenness was calculated using Simpson’s evenness index, and phylogenetic diversity was calculated using Faith’s *D* index. Gray symbols are the observed data, and black symbols represent means ± 95% confidence intervals.

**FIG 4:**
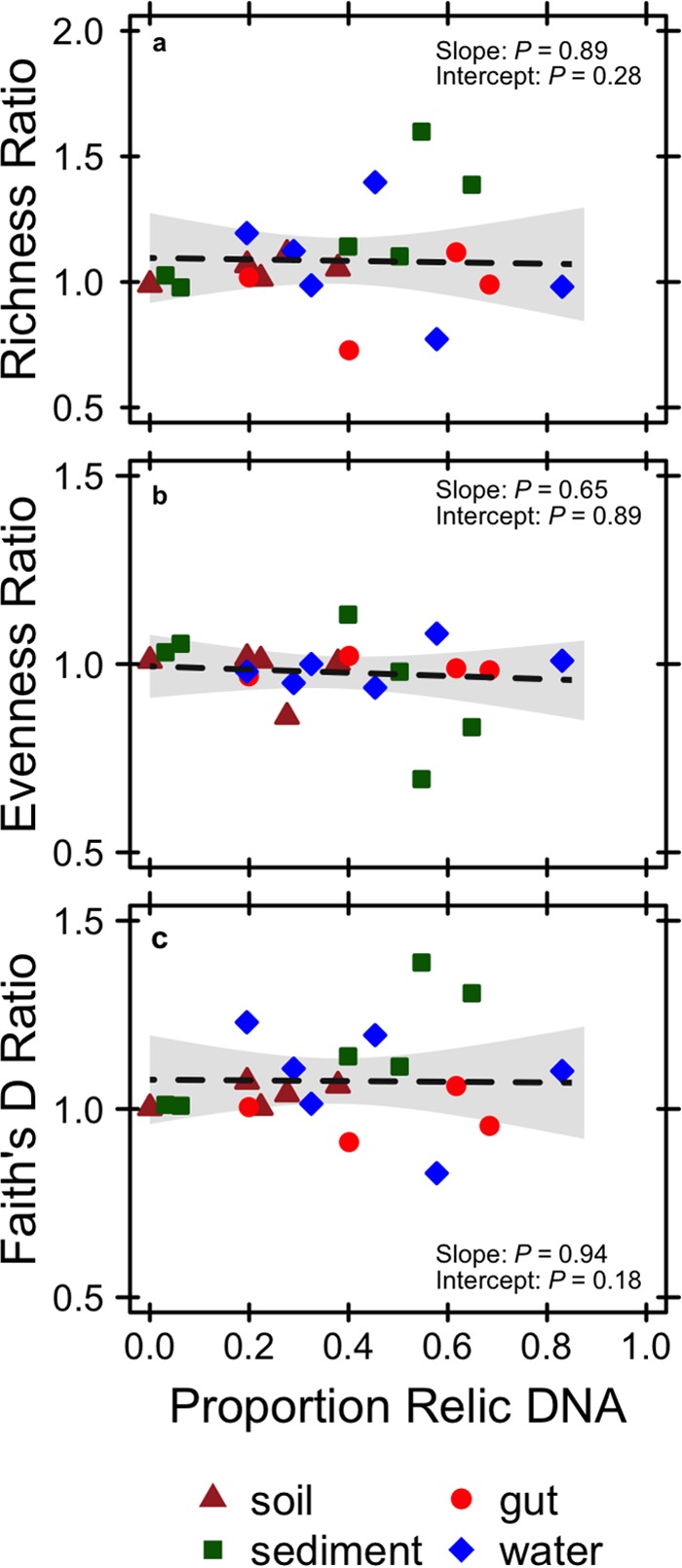
Linear regressions testing for the effect of relic DNA on measures of alpha-diversity. See the text for descriptions of how diversity metrics and diversity ratios were calculated. When included in the regression model as an indicator variable, ecosystem type had no effect on the slopes or intercepts of the regressions (*P* ≥ 0.26).

Despite accounting for a substantial portion of the total DNA (Fig. 2), relic DNA had a minimal effect on estimates of richness, evenness, phylogenetic diversity (PD), or the species abundance distribution (SAD) based on 16S rRNA gene sequencing (Table S1). We standardized the effect size of these alpha-diversity metrics on a per-sample basis as a ratio (total/intact), where values of >1 represent overestimation bias and values of <1 represent underestimation bias. Relic DNA had no effect on the diversity ratios based on the observation that the 95% confidence intervals overlapped with 1.0. Furthermore, the 95% confidence intervals of the diversity ratios overlapped across ecosystem types, indicating that the contribution of relic DNA to all measures of diversity was generally low irrespective of the microbial habitat (Fig. 3). We used Kolmogorov-Smirnov tests to determine whether relic DNA altered the SAD. For each paired sample, we tested if the distribution of abundances for taxa in the total DNA pool was different from distribution of abundances for taxa in the intact DNA pool. From these analyses, we conclude that the abundance distributions of taxa are similar and not affected by ecosystem type (*P* ≥ 0.3), which suggests that patterns of commonness and rarity are not influenced by relic DNA. Finally, using indicator variable multiple regression, we evaluated whether the magnitude of bias increased with relic DNA pool size, a predicted outcome from some of our sampling- and process-based simulations (Fig. 1b and c). For all diversity ratios (richness, evenness, and PD), the slopes were not different from zero (*P* ≥ 0.65), the intercepts were not different from 1.0 (*P* ≥ 0.65), and the estimates were not affected by ecosystem type (*P* ≥ 0.26) (Fig. 4). Moreover, only a very small amount of variation (*R*^2^ ≤ 0.01) was explained by relic DNA and ecosystem type in the regression models.

10.1128/mBio.00637-18.8 TABLE S1  Proportion of relic DNA in samples from different ecosystem types. Download TABLE S1, PDF file, 0.1 MB. Copyright © 2018 Lennon et al. 2018 Lennon et al. This content is distributed under the terms of the Creative Commons Attribution 4.0 International license.

### (iii) Contribution of relic DNA to community composition.

**FIG 5:**
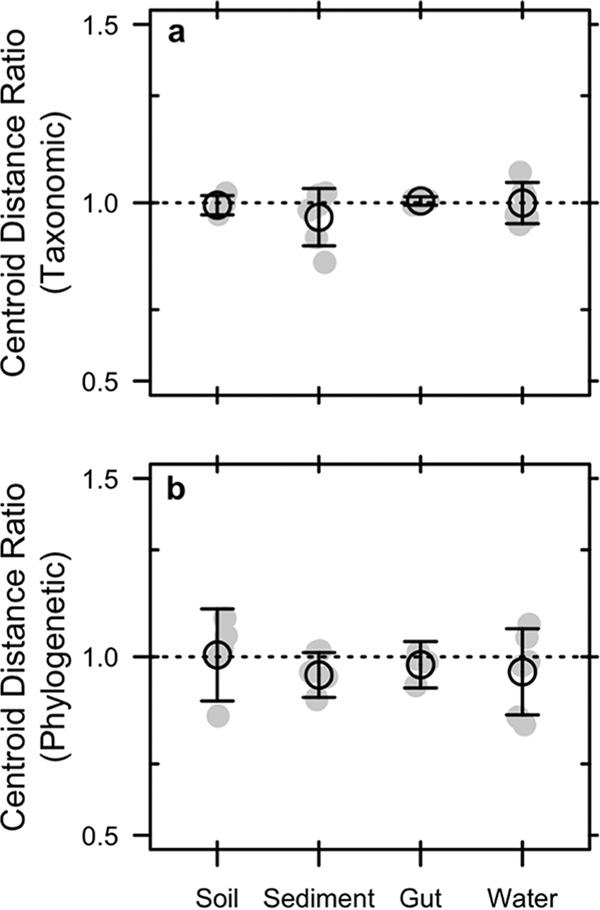
Effect of relic DNA on the among-sample (beta) bacterial diversity in different ecosystem types. (a and b) We tested for the effects of bias caused by relic DNA by calculating a beta-diversity ratio based on centroid distances. Centroid distances were estimated after performing principal-coordinate analyses (PCoAs) using taxonomic (a) and phylogenetic (b) distance metrics (Bray-Curtis and UniFrac, respectively). The centroid distance ratio was calculated on each sample within an ecosystem type and reflects the composition of the total DNA pool (intact + relic) relative to the intact DNA pool. Relic DNA had no effect on beta-diversity for any of the ecosystem types sampled. Gray symbols are the observed data, and black symbols represent means ± 95% confidence intervals.

**FIG 6:**
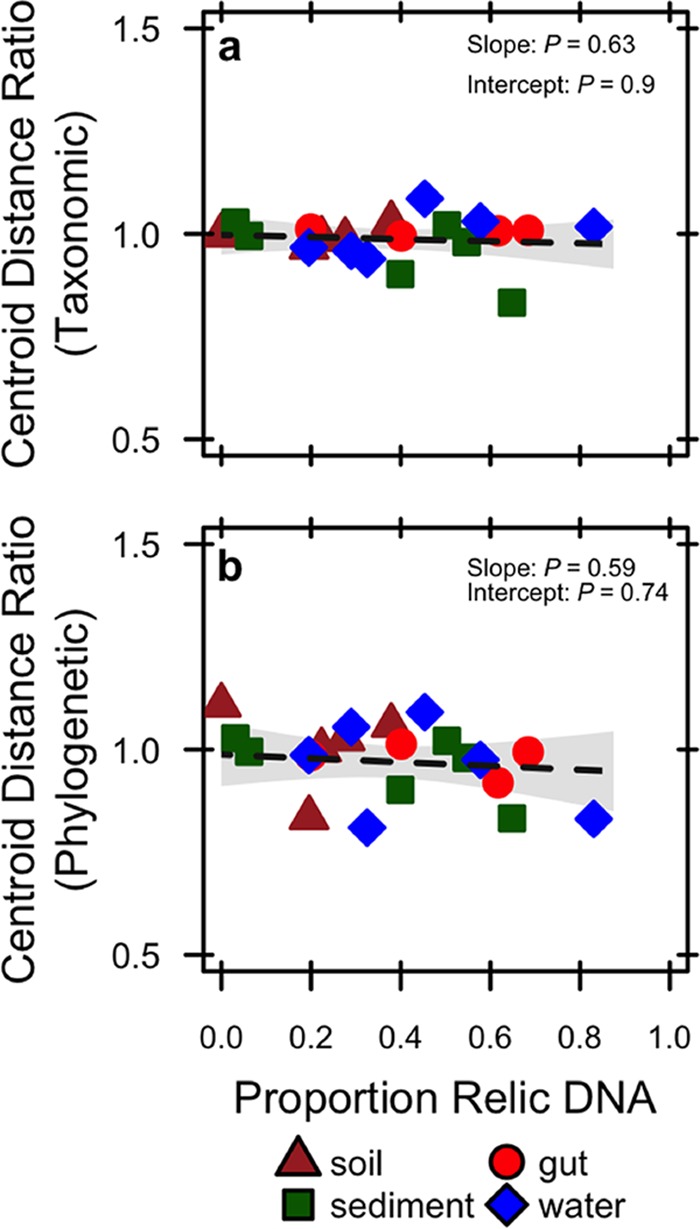
Linear regression testing for effect of relic DNA on measures of beta-diversity using taxonomic distances calculated using the Bray-Curtis method (a) and phylogenetic distances calculated using UniFrac (b). See Materials and Methods and Fig. S6 for a description of how centroid distance ratios were calculated. When included in the regression model as an indicator variable, ecosystem type had no effect on the slopes or intercepts of the regressions (*P* ≥ 0.29).

Based on 16S rRNA gene sequencing of the intact and total DNA pools, relic DNA had a minimal effect on the compositional (beta) diversity of bacterial communities within and across ecosystem types. First, the intact and total DNA pools were significantly and very highly correlated with one another when we performed a taxonomically based (Bray-Curtis distance) Mantel test (*P* = 0.001, *r* = 0.959) and a phylogenetically based (UniFrac distance) Mantel test (*P* = 0.001, *r* = 0.996). Second, we tested for the effect of relic DNA on bacterial community composition for the intact and total DNA pools using a modified beta-dispersion metric (22). Specifically, we calculated centroid distance ratios, which directly compared the dispersion between pairs of DNase-treated and DNase-control subsamples (see Fig. S6). With this approach, if the centroid distance ratio was >1, we concluded that relic DNA inflated beta-diversity; if the distance ratio was <1, we concluded that relic DNA homogenized beta-diversity. We found that relic DNA had no effect on the centroid distance ratios based on the observation that the 95% confidence intervals overlapped with 1.0 (Fig. 5 and S6). Furthermore, the 95% confidence intervals for the centroid distance ratios overlapped across ecosystem types, indicating that the contribution of relic DNA to taxonomic and phylogenetic beta-diversity was low irrespective of the microbial habitat. Last, we used indicator variable multiple regression to test the prediction that bias in estimates of community composition would increase with increasing relic DNA pool size (see Fig. S1). The proportion of relic DNA in a sample had no statistical effect on the slopes for centroid distance ratios regardless of whether they were calculated using taxonomic (*P* = 0.63) or phylogenetic (*P* = 0.59) distance matrices. Moreover, the intercepts for these regression relationships were not different from 1.0 (*P* ≥ 0.74), and the amount of explained variation was very small (*R*^2^ ≤ 0.09), suggesting that the overall effect of relic DNA on beta-diversity was negligible (Fig. 6). The parameter estimates from the regression analyses were not affected by ecosystem type (*P* ≥ 0.29).

## DISCUSSION

Rarely, if ever, are biological communities completely censused. As a result, estimates of diversity are often based on incomplete sampling, which introduces uncertainty and potential bias (23). For example, microbiologists using cultivation-independent approaches commonly estimate the diversity of a community using hundreds of thousands of sequences (10^5^) from samples that contain in excess of a billion individuals (10^9^). Diversity estimation of microbial communities is further complicated by the accumulation and persistence of relic DNA, a pool of sequences that may not accurately reflect the composition of viable microorganisms. We developed a set of models that capture the fundamental processes regulating relic DNA dynamics along with the effects of sampling from a joint species abundance distribution (SAD) that contains sequences from both the relic and intact DNA pools. Our models reveal that two criteria are required in order for relic DNA bias to emerge. First, the relic DNA pool must be large enough so that it diminishes the probability of sampling sequences from the intact DNA pool. Second, the relic SAD must be distinct from the intact SAD. If these conditions are not satisfied, relic DNA should have a minimal effect on estimates of biodiversity. We then explored the model’s expectations by analyzing the intact and total (relic + intact) DNA in a range of ecosystems where relic DNA dynamics are known to vary. Despite its making up a large portion of the total DNA, we found that relic DNA had minimal effect on estimates of taxonomic and phylogenetic diversity, consistent with model scenarios where relic DNA degradation rates are equivalent among species.

Despite sampling a range of habitats with attributes that affect relic DNA turnover, our data suggest that estimates of taxonomic and phylogenetic diversity remained relatively unbiased by relic DNA (Fig. 2 and 3) even when it accounted for >80% of the total DNA (Fig. 4 and 6). Similar findings have been reported elsewhere. From a survey of North American soils, no bias was detected for taxonomic richness in 30% of bacterial samples (7/31) and 55% of fungal samples (17/31), even when relic DNA made up a substantial portion of the total DNA pool (21). Likewise, more than 90% of the bacterial DNA recovered from porcine lung tissues was sensitive to DNase, yet diversity values in the enzymatically treated (i.e., DNase) and untreated samples were the same, indicating that large relic DNA pools do not necessarily obscure estimates of microbial diversity (20). Such findings are supported by our modeling results: relic DNA has no effect on estimates of microbial diversity when the SADs of the relic and intact DNA pools are equivalent (Fig. 1b). When this assumption is met, the abundance distributions of relic and intact DNA pools should be identical. Therefore, sampling from the relic DNA pool should not bias estimates of diversity in the intact DNA pool, even if relic DNA makes up a large portion of the total DNA pool (Fig. 1b).

Since bias has been documented in some studies, the important question becomes under what conditions are we likely to see differences in the SADs of the relic and intact DNA pools? One leading hypothesis is that relic DNA can be protected from degradation inside aggregates, biofilms, or other complexes that reduce the contact rate of nucleic acids and extracellular enzymes. Importantly, our simulations reveal that protection can increase bias only if it alters relic DNA degradation in a species-specific manner. While most studies have emphasized the potential for protection to overestimate diversity, our model predicts the opposite (Fig. 1b). Protection creates a less even relic SAD that results in the increased dominance of relic sequences associated with a few species. Assuming a large relic DNA pool, preferential sampling of these protected sequences will lead to the underestimation of richness, which has been observed but only in a few samples (21).

In contrast, we identified at least one scenario where relic DNA can create overestimation bias. Microscale heterogeneity in structured habitats can lead to nonuniform distributions of microorganisms and their metabolic activities. Our simulations indicate that the resulting “hot spots” can degrade relic DNA sequences in a density-dependent manner, resulting in a more even relic SAD. Although not statistically significant, sediment samples trended toward positive bias for richness and PD (Fig. 3), which could potentially reflect “hot spots” of relic DNA degradation. There are other situations where inflation bias could potentially arise, including when species in the regional pool disperse into habitats for which they are poorly adapted. For example, our simulations indicate that when poorly adapted immigrants immediately die, they can enrich the relic DNA pool with allochthonous sequences that are dissimilar to sequences found in the local community and thereby influence estimates of diversity (see Fig. S7 in the supplemental material). A similar effect could arise when dead bacteria are transported across ecosystem boundaries, a phenomenon that occurs, for example, when marine snow is exported from surface waters to marine sediments (24). More generally, bias may arise under nonequilibrium conditions where community turnover of the intact DNA pool is faster than turnover of the relic DNA pool. Any abiotic or biotic perturbation that removes a substantial amount of living biomass could result in transient divergence in the composition of sequences in the intact and relic DNA pools. For example, virulent phage can rapidly reduce the abundance of bacterial prey and in the process release a large pulse of dissolved DNA into the environment (25). In this way, relic DNA might distort estimates of temporal stability in microbial communities. Last, although not fully explored here, shifts in rank abundance of taxa that are independent of parameters describing the SAD could also result in biased estimates of microbial diversity. In sum, there are ways to deviate from neutral expectations about the degradation of relic DNA, which should lead to biased estimates of microbial diversity. And, of course, many of the processes described above can operate simultaneously, which may amplify or dampen the effects of relic DNA bias. Data from our study suggest that such conditions may not be prevalent, but additional sampling across an even broader range of ecosystems is needed to determine whether or not relic DNA bias is a major concern for estimating biodiversity.

Our understanding of the microbial biosphere has been transformed by the development and application of molecular biology-based cultivation-independent techniques. The ability to rapidly obtain millions of gene sequences and transcripts from a range of environments has yielded valuable insight into the processes that regulate community assembly and function (26, 27) and has also paved the way for the discovery of new metabolisms (28), tests for unifying patterns of biodiversity (29), and an updated tree of life (30). There are limitations, however, associated with culture-independent techniques, which include inefficient nucleic acid extraction methods (31) and “universal” primers that overrepresent some taxonomic groups while overlooking others (32). Sequencing of relic DNA is another important concern, which can potentially lead to the overestimation or underestimation of microbial diversity. However, this bias requires the decoupling of processes that regulate the compositional turnover of the relic and intact DNA pools. While some recent evidence suggests that this can arise (21), our findings suggest that at least in some ecosystems, relic DNA appears to contribute minimally to the characterization of microbial community structure.

## MATERIALS AND METHODS

### Theoretical framework for relic DNA dynamics and diversity. (i) Sampling model.

We used a set of sampling-based simulations to explore how the mixing of intact and relic DNA influences estimates of diversity. For each simulation, we defined a regional species pool consisting of 10,000 taxa with a lognormal abundance distribution (33). The intact community consisted of 1,000,000 individuals randomly sampled from the regional species pool. To evaluate the hypothesis that the species abundance distributions (SADs) must be different between the intact and relic DNA in order for bias to arise, we altered the evenness of the regional pool from which the relic community was sampled by changing the scale parameter of the lognormal distribution. To decrease the evenness of the relic DNA pool, we increased the scale parameter from 0.98 to 1.8. To increase the evenness of the relic DNA pool, we decreased the parameter from 0.98 to 0.25. After mixing the intact and relic communities at the defined proportions ranging from 0.01 to 0.96, we rarified the total community to 10,000 observations before estimating richness and compositional dissimilarity of DNA pools in a simulated sample. We represent the effects of relic DNA bias for a given sample using diversity ratios (total DNA/intact DNA) for richness and Bray-Curtis distances. For example, if relic DNA results in no bias, then the diversity ratio for a sample will equal 1.0. However, if relic DNA inflates diversity, then the diversity ratio will be greater than 1. If diversity ratios are less than 1, then relic DNA should underestimate the true diversity of a sample.

### (ii) Process-based model.

We developed a set of stochastic simulations to explore how the processes regulating relic DNA dynamics can give rise to bias in the estimation of microbial diversity. For each simulation, we defined a regional species pool consisting of 4,000 taxa with a lognormal abundance distribution (33). We initiated an intact community by randomly sampling 20,000 individuals from the regional species pool. Subsequent dynamics were controlled by the processes depicted in our conceptual model (Fig. 1a). Specifically, we simulated immigration by sampling *j* individuals from the regional species pool and adding them to the intact community. We simulated birth by randomly selecting *I* × *r* individuals from the intact community and adding them to the intact community again, where *I* is the size of the intact community and *r* is the per capita birthrate. We simulated death by selecting (*I* × *m*) + *j* individuals from the intact community and moving them to the relic community (*R*), where *m* is the per capita mortality rate. Finally, we simulated degradation by selecting (*R* × *d*) + *j* individuals from the relic community and removing them, where *d* is the per capita decay rate of relic DNA.

With the process-based model in place, we investigated three scenarios that we hypothesized could affect relic DNA dynamics and its effects on diversity estimates. First, we considered a neutral scenario. At each time step, we simulated degradation by randomly selecting relic DNA sequences and removing them. Second, we considered the scenario where some relic DNA sequences have lower rates of degradation owing to processes such as physical protection. Each species was randomly assigned a degradation susceptibility probability from a beta distribution. The beta distribution is a continuous probability distribution bound by 0 and 1 with two shape parameters. We selected shape parameters (α = 0.7, β = 0.7) that resulted in a U-shaped distribution of susceptibilities such that values close to 0 had low probabilities of degradation and values close to 1 had high probabilities of degradation. Susceptibility probabilities were randomly assigned to species for each model iteration and were used to weight the probability of selecting relic DNA sequences for degradation at each time step. Last, we considered the scenario where there are “hot spots” of relic DNA degradation, reflecting the clumped distribution of microbial taxa and their associated metabolic activities. At each time step, we calculated density-dependent selection probabilities by weighting the probability of selecting an individual from the relic community by its species abundance. Therefore, relic DNA sequences belonging to more abundant species would have a higher probability of degrading. For each of the three scenarios, we ran each simulation for 10,000 time steps. We used a constant immigration rate (*j* = 2,000) to maintain intact community diversity and used equal and constant birth and mortality rates (*b* and *m* = 0.1) to maintain intact community size. Likewise, we accounted for immigration during death and degradation to prevent uncontrolled growth. For each set of simulations, we used a range of decay rates (*d*), which yielded relic DNA proportions between 0.05 and 0.95 according to the following equation: *d* = *m*/*p* − *m*, where *p* is the target proportion and *m* is the fixed mortality rate (see the supplemental material for derivation). At the end of each simulation, we determined the effect of relic DNA on diversity estimates by comparing the total community (intact + relic) with the intact community. All simulations and estimations were performed in the R statistical environment (v 3.3.2) (34) using the “vegan” package as well as custom functions.

### Empirical assessment of relic DNA abundance and effects on diversity estimation. (i) Sample collection and DNA pools.

We collected samples from a diverse set of environmental and host-associated ecosystems. First, we sampled sediments and surface water from lakes near the Michigan State University W. K. Kellogg Biological Station (KBS) in Hickory Corners, MI. Soils were sampled from the main sites and surrounding areas at the KBS Long-Term Ecological Research site (35). We also collected fresh feces as representative gut samples from cows, dogs, horses, rabbits, and humans. In each of the ecosystem types, we obtained samples from 6 to 8 independent sites. In the laboratory, we applied a DNase treatment to half of a well-homogenized sample to remove relic DNA (Fig. S2). This enzymatic removal procedure is based on methods that have previously been used to quantify relic DNA in marine sediments (36, 37), host tissue (36, 37), and drinking-water biofilms (36, 37). DNase is effective at removing not only extracellular DNA but also DNA contained inside dead cells while not compromising the integrity of living cells (36, 37). After assessing the optimal DNase concentration (Fig. S3), we determined that the DNase method was extremely efficient (98%) at removing a spike of 16S rRNA into a soil sample (Fig. S4). Thus, we refer to the DNA remaining following enzymatic treatment as “intact DNA” and assume that it was derived from viable cells. “Total DNA” refers to the DNA in the other half of the sample that was not treated with DNase (negative control), which reflects the sum of intact DNA and relic DNA.

10.1128/mBio.00637-18.2 FIG S2  Sample processing to quantify the proportion of relic DNA. Download FIG S2, PDF file, 0.1 MB. Copyright © 2018 Lennon et al. 2018 Lennon et al. This content is distributed under the terms of the Creative Commons Attribution 4.0 International license.

10.1128/mBio.00637-18.3 FIG S3  Effect of DNase I concentration on the estimated proportion of relic DNA. Download FIG S3, PDF file, 0.1 MB. Copyright © 2018 Lennon et al. 2018 Lennon et al. This content is distributed under the terms of the Creative Commons Attribution 4.0 International license.

10.1128/mBio.00637-18.4 FIG S4  Efficiency of quantifying relic DNA. Download FIG S4, PDF file, 0.1 MB. Copyright © 2018 Lennon et al. 2018 Lennon et al. This content is distributed under the terms of the Creative Commons Attribution 4.0 International license.

10.1128/mBio.00637-18.5 FIG S5  Principal-coordinate analysis (PCoA) of bacterial communities. Download FIG S5, PDF file, 0.1 MB. Copyright © 2018 Lennon et al. 2018 Lennon et al. This content is distributed under the terms of the Creative Commons Attribution 4.0 International license.

For aquatic samples, we filtered 250 ml lake water through a 47-mm 0.2-µm Supor polyethersulfone (PES) membrane filter using a 10-mm Hg vacuum. We cut the filter in half and randomly assigned one half of the filter to a DNase treatment and used the other half as the control. We then rolled up each piece of filter using sterile forceps and inserted it into a 2-ml centrifuge tube containing 1.5 ml of pH 7.3 phosphate-buffered saline (PBS). After vortexing at room temperature for 5 min, we removed the filter from the tube and centrifuged for 5 min at 10,000 × *g*. We then discarded the supernatant and resuspended the pellet in 375 µl of PBS, which was transferred to a 2-ml centrifuge tube. For nonaqueous materials (soil, sediments, and feces), we directly added 0.25 g of well-homogenized sample to a 2-ml centrifuge tube. At this stage in the procedure, aquatic and nonaquatic samples were identically processed. For each sample in its 2-ml centrifuge tube, we added DNase digestion buffer, which consisted of 382.5 µl of Nanopure water, 5 µl of 1 M MgCl_2_, 2.5 µl of bovine serum albumin (10 mg/ml), and 50 µl of 0.5 M Tris-HCl (pH 7.5). For subsamples treated with DNase, we added 40 µl of a 10-U/µl stock of DNase I (Roche catalog no. 04536282001) and 20 µl of Nanopure water, resulting in a 500-µl final working volume with an 0.8-U/µl DNase concentration. For untreated subsamples, we substituted 40 µl of Nanopure water for DNase solution. For each sample, we measured pH using a micro-pH probe (Orion 9110DJWP; Thermo Scientific) and adjusted the pH to 7.3 to 7.7, which is in the optimum range for DNase. We then incubated the samples horizontally on a shaker table at 37°C for 60 min. Following this, we transferred each sample to a 15-ml Falcon tube containing 1 ml of 1× hexadecyltrimethylammonium bromide (CTAB) buffer, which consisted of one part solution 1 (1 g CTAB plus 0.58 g NaCl in 10 ml of Nanopure water) and one part solution 2 (0.58 g NaCl plus 0.82 K_2_HPO_4_ plus 0.04 KH_2_PO_4_ in 10 ml of Nanopure water). We then added 25 µl of 0.5 M EDTA to each tube and vortexed. We stopped the DNase reaction by incubating the tubes at 75°C for 10 min. We began DNA extraction by adding 1 ml of phenol-chloroform-isoamyl alcohol (25:24:1) to the 2-ml tube, which was then vortexed for 10 min. This was followed by centrifugation at 7,100 × *g* for 10 min. We transferred the top aqueous layer to a 15-ml Falcon tube, combined it with an equal volume of chloroform-isoamyl alcohol (24:1), and then vortexed the mixture. After centrifuging the Falcon tubes at 7,100 × *g* for 5 min, we transferred 400 µl of the top aqueous layer to a new 2-ml centrifuge tube. For cleanup, we used the Mo Bio PowerLyzer PowerSoil DNA isolation kit starting from step 9. At step 21 of the DNA isolation kit, we eluted the DNA in 50 µl of solution C6 and centrifuged it for 1 min at 10,000 × *g*. We stored extracted DNA at −20°C for short-term storage or at −80°C for long-term storage.

### (ii) Contribution of relic DNA to bacterial abundance.

We used 16S rRNA gene copy numbers generated from quantitative PCR (qPCR) assays to estimate the proportion of relic DNA in a sample as 1 − (intact DNA/total DNA). Following procedures described elsewhere (38), we performed qPCR assays where 30-µl reaction mixtures contained 1 µl of DNA template, 0.5 µl of each primer (10 µM), 14.5 µl of nuclease-free H_2_O, and 13.5 µl of 5 Prime 2.5× RealMasterMix SYBR ROX. We amplified a 200-bp fragment of the 16S rRNA gene with Eub338 (forward) and Eub518 (reverse) primers (39). PCR assays were performed with an Eppendorf Mastercycler Realplex^2^ system using previously reported thermal cycle conditions (39). We generated qPCR standards from bacterial genomic DNA (*Micrococcus* sp.) using the TOPO TA cloning kit (Invitrogen). We extracted plasmids from transformed cells (40) and used the M13 forward and reverse primers to generate PCR products. The PCR products were quantified using a noise-band threshold and then used to generate a standard curve capturing a range of 10^2^ to 10^7^ gene copies/µl. The coefficients of determination (*r*^2^) for our assays ranged from 0.96 to 0.99, while amplification efficiencies fell between 0.93 and 0.99. Based on melting curve analyses, we found no evidence for primer dimers. All unknown samples, no-template controls, and standards were run in triplicate on every plate. The mean coefficient of variation (standard deviation/mean) for our 16S rRNA qPCR assay was 0.16.

### (iii) Contribution of relic DNA to bacterial diversity.

We estimated the contribution of relic DNA to bacterial diversity using high-throughput sequencing of the 16S rRNA gene. Specifically, we amplified the V4 region of the 16S rRNA gene from the intact and total DNA pools of each sample using bar-coded primers (515F and 806R) designed to work with the Illumina MiSeq platform (41). We cleaned the sequence libraries using the AMPure XP purification kit, quantified the resulting products using the QuantIt PicoGreen kit (Invitrogen), and pooled libraries at equal molar ratios (final concentration, 20 ng per library). We then sequenced the pooled libraries with the Illumina MiSeq platform using paired-end reads (Illumina Reagent kit v2, 500-reaction kit) at the Indiana University Center for Genomics and Bioinformatics Sequencing Facility. Paired-end raw 16S rRNA sequence reads were assembled into contigs using the Needleman algorithm (42). We obtained a total of 12,916,632 16S rRNA sequences. After quality trimming with a moving average quality score (window, 50 bp; minimum quality score, 35), we aligned the sequences to the Silva Database (version 123) using the Needleman algorithm. Chimeric sequences were removed using the UCHIME algorithm (43). After this filtering, there was an average (± standard error of the mean [SEM]) of 222,701 ± 9,560 sequences per site. We created operational taxonomic units (OTUs) by first splitting the sequences based on taxonomic class (using the RDP taxonomy) and then binning sequences into OTUs based on 97% sequence similarity. Our depth of sequencing led to a high degree of coverage across samples (minimum Good’s coverage = 0.98). For phylogenetic analysis, we picked representative sequences for each OTU by using the most abundant unique sequence. We used FastTree (44) to generate a phylogenetic tree from the representative sequences using the generalized time-reversible model of nucleotide evolution. We calculated phylogenetic distances using weighted UniFrac distances (45). All initial sequence processing was completed using the software package mothur (version 1.38.1) (46).

We estimated the effects of relic DNA on alpha-diversity by calculating richness, evenness, and phylogenetic diversity for the intact and total DNA pools within a sample. To estimate the number of OTUs (richness), we used a resampling approach that subsampled each sample to an equal number of sequences per sample and summed the number of OTUs that were represented (47). Briefly, we subsampled to 30,000 observations, resampled 999 additional times, and then calculated the average richness estimates (± SEM) for each sample. To estimate the equitability in abundance among taxa in a sample (evenness), we used the same resampling approach and calculated average evenness estimates (± SEM) using Simpson’s evenness index (48). To test whether relic DNA affected the phylogenetic diversity within a sample, we subsampled communities to 30,000 observations and then calculated Faith’s *D* statistic, which sums the branch lengths for each species found in a sample from the root to the tip of the phylogenetic tree (49). In addition, we used two-sample Kolmogorov-Smirnov tests to determine whether or not the abundance distribution of taxa in the intact pool was different from that in the total pool for paired samples. We then calculated the average *D* statistics and *P* values from the Kolmogorov-Smirnov tests to evaluate the effect of relic DNA across ecosystem types. All estimations were performed in the R statistical environment (v 3.3.2) (34) using the “vegan,” “ape,” “ade4,” “picante,” and “plyr” packages, along with custom functions.

We estimated the effects of relic DNA on beta-diversity by comparing the taxonomic and phylogenetic diversity of bacterial communities in the intact and total DNA pools. First, we conducted a principal-coordinate analysis (PCoA) on log_10_-transformed relative abundance data to visualize the effects of relic DNA removal (via DNase treatment) on bacterial community composition within and among ecosystem types. The PCoA was performed with Bray-Curtis and UniFrac distances to assess taxonomic and phylogenetic effects, respectively. In addition, we used permutational multivariate analysis of variance (PERMANOVA) to test for differences in taxonomic and phylogenetic composition based on ecosystem type for the total DNA pool. Second, we conducted a Mantel test to assess the correlation between the community resemblance matrices (either Bray-Curtis or UniFrac) represented by the intact and total DNA pools. Last, we tested whether relic DNA altered beta-diversity within an ecosystem type by comparing centroid distances. To calculate this metric of sample dispersion, we determined the centroid from a PCoA with either Bray-Curtis or UniFrac distances for the total DNA pool for all sites within a given ecosystem type. We then measured the Euclidean distances between the centroid and all samples (total and intact) to determine the centroid distances (see Fig. S6 for more detail).

10.1128/mBio.00637-18.6 FIG S6  Centroid distance ratios used to quantify effects of relic DNA on beta-diversity. Download FIG S6, PDF file, 0.1 MB. Copyright © 2018 Lennon et al. 2018 Lennon et al. This content is distributed under the terms of the Creative Commons Attribution 4.0 International license.

### Data and software availability.

All code and data used in this study can be found in a public GitHub repository (https://www.github.com/LennonLab/relicDNA) and the NCBI SRA (BioProject PRJNA464404).

10.1128/mBio.00637-18.9 TABLE S2  Estimates of diversity within samples from different ecosystem types. Download TABLE S2, PDF file, 0.1 MB. Copyright © 2018 Lennon et al. 2018 Lennon et al. This content is distributed under the terms of the Creative Commons Attribution 4.0 International license.

10.1128/mBio.00637-18.7 FIG S7  Effect of relic DNA immigration on richness estimates. Download FIG S7, PDF file, 0.1 MB. Copyright © 2018 Lennon et al. 2018 Lennon et al. This content is distributed under the terms of the Creative Commons Attribution 4.0 International license.

## References

[1] Redfield RJ 1993 Genes for breakfast: the have-your-cake-and-eat-it-too of bacterial transformation. J Hered 84:400–404. doi:10.1093/oxfordjournals.jhered.a111361. 8409360

[2] Dell’Anno A, Danovaro R 2005 Extracellular DNA plays a key role in deep-sea ecosystem functioning. Science 309:2179–2179. doi:10.1126/science.1117475. 16195451

[3] Lennon JT 2007 Diversity and metabolism of marine bacteria cultivated on dissolved DNA. Appl Environ Microbiol 73:2799–2805. doi:10.1128/AEM.02674-06. 17337557 PMC1892854

[4] Klein DA 2007 Microbial communities in nature: a postgenomic perspective. Microbe 2:591–595. doi:10.1128/microbe.2.591.1.

[5] Luna GM, Manini E, Danovaro R 2002 Large fraction of dead and inactive bacteria in coastal marine sediments: comparison of protocols for determination and ecological significance. Appl Environ Microbiol 68:3509–3513. doi:10.1128/AEM.68.7.3509-3513.2002. 12089035 PMC126761

[6] Lennon JT, Jones SE 2011 Microbial seed banks: the ecological and evolutionary implications of dormancy. Nat Rev Microbiol 9:119–130. doi:10.1038/nrmicro2504. 21233850

[7] Vorkapic D, Pressler K, Schild S 2016 Multifaceted roles of extracellular DNA in bacterial physiology. Curr Genet 62:71–79. doi:10.1007/s00294-015-0514-x. 26328805 PMC4723616

[8] Levy-Booth DJ, Campbell RG, Gulden RH, Hart MM, Powell JR, Klironomos JN, Pauls KP, Swanton CJ, Trevors JT, Dunfield KE 2007 Cycling of extracellular DNA in the soil environment. Soil Biol Biochem 39:2977–2991. doi:10.1016/j.soilbio.2007.06.020.

[9] Torti A, Lever MA, Jørgensen BB 2015 Origin, dynamics, and implications of extracellular DNA pools in marine sediments. Mar Genomics 24:185–196. doi:10.1016/j.margen.2015.08.007. 26452301

[10] Paul JH, Jeffrey WH, David AW, DeFlaun MF, Cazares LH 1989 Turnover of extracellular DNA in eutrophic and oligotrophic freshwater environments of southwest Florida. Appl Environ Microbiol 55:1823–1828. 16347976 10.1128/aem.55.7.1823-1828.1989 PMC202957

[11] Dell’Anno A, Corinaldesi C 2004 Degradation and turnover of extracellular DNA in marine sediments: ecological and methodological considerations. Appl Environ Microbiol 70:4384–4386. doi:10.1128/AEM.70.7.4384-4386.2004. 15240325 PMC444808

[12] DeFlaun MF, Paul JH 1989 Detection of exogenous gene sequences in dissolved DNA from aquatic environments. Microb Ecol 18:21–28. doi:10.1007/BF02011693. 24196018

[13] DeFlaun MF, Paul JH, Jeffrey WH 1987 Distribution and molecular weight of dissolved DNA in subtropical estuarine and oceanic environments. Mar Ecol Prog Ser 38:65–73. doi:10.3354/meps038065.

[14] Beebee TJC 1993 Identification and analysis of nucleic acids in natural freshwaters. Sci Total Environ 135:123–129. doi:10.1016/0048-9697(93)90282-B.

[15] Romanowski G, Lorenz MG, Wackernagel W 1991 Adsorption of plasmid DNA to mineral surfaces and protection against DNase I. Appl Environ Microbiol 57:1057–1061. 1647748 10.1128/aem.57.4.1057-1061.1991 PMC182845

[16] Schwechheimer C, Kuehn MJ 2015 Outer-membrane vesicles from Gram-negative bacteria: biogenesis and functions. Nat Rev Microbiol 13:605–619. doi:10.1038/nrmicro3525. 26373371 PMC5308417

[17] DeAngelis KM, Lindow SE, Firestone MK 2008 Bacterial quorum sensing and nitrogen cycling in rhizosphere soil. FEMS Microbiol Ecol 66:197–207. doi:10.1111/j.1574-6941.2008.00550.x. 18721146

[18] Emerson JB, Adams RI, Román CMB, Brooks B, Coil DA, Dahlhausen K, Ganz HH, Hartmann EM, Hsu T, Justice NB, Paulino-Lima IG, Luongo JC, Lymperopoulou DS, Gomez-Silvan C, Rothschild-Mancinelli B, Balk M, Huttenhower C, Nocker A, Vaishampayan P, Rothschild LJ 2017 Schrodinger’s microbes: tools for distinguishing the living from the dead in microbial ecosystems. Microbiome 5:86. doi:10.1186/s40168-017-0285-3. 28810907 PMC5558654

[19] Sun J, Wang P, Shi Y 2016 High fat diet decrease diversity of extracellular DNA of mice gut microbiota. FASEB J 30(Suppl):lb204.

[20] Pezzulo AA, Kelly PH, Nassar BS, Rutland CJ, Gansemer ND, Dohrn CL, Costello AJ, Stoltz DA, Zabner J 2013 Abundant DNase I-sensitive bacterial DNA in healthy porcine lungs and its implications for the lung microbiome. Appl Environ Microbiol 79:5936–5941. doi:10.1128/AEM.01752-13. 23872563 PMC3811377

[21] Carini P, Marsden PJ, Leff JW, Morgan EE, Strickland MS, Fierer N 2016 Relic DNA is abundant in soil and obscures estimates of soil microbial diversity. Nat Microbiol 2:16242. doi:10.1038/nmicrobiol.2016.242. 27991881

[22] Anderson MJ, Ellingsen KE, McArdle BH 2006 Multivariate dispersion as a measure of beta diversity. Ecol Lett 9:683–693. doi:10.1111/j.1461-0248.2006.00926.x. 16706913

[23] Magurran AE 2004 Measuring biological diversity. Wiley-Blackwell, Oxford, United Kingdom.

[24] Azam F, Long RA 2001 Oceanography: sea snow microcosms. Nature 414:495–498. doi:10.1038/35107174. 11734832

[25] Corinaldesi C, Tangherlini M, Luna GM, Dell’Anno A 2014 Extracellular DNA can preserve the genetic signatures of present and past viral infection events in deep hypersaline anoxic basins. Proc Biol Sci 281:20133299. doi:10.1098/rspb.2013.3299. PMC4027399 24523277

[26] Lindström ES, Langenheder S 2012 Local and regional factors influencing bacterial community assembly. Environ Microbiol Rep 4:1–9. doi:10.1111/j.1758-2229.2011.00257.x. 23757223

[27] Aanderud ZT, Jones SE, Fierer N, Lennon JT 2015 Resuscitation of the rare biosphere contributes to pulses of ecosystem activity. Front Microbiol 6:24. doi:10.3389/fmicb.2015.00024. 25688238 PMC4311709

[28] Béjà O, Aravind L, Koonin EV, Suzuki MT, Hadd A, Nguyen LP, Jovanovich SB, Gates CM, Feldman RA, Spudich JL, Spudich EN, DeLong EF 2000 Bacterial rhodopsin: evidence for a new type of phototrophy in the sea. Science 289:1902–1906. doi:10.1126/science.289.5486.1902. 10988064

[29] Locey KJ, Lennon JT 2016 Scaling laws predict global microbial diversity. Proc Natl Acad Sci U S A 113:5970–5975. doi:10.1073/pnas.1521291113. 27140646 PMC4889364

[30] Hug LA, Baker BJ, Anantharaman K, Brown CT, Probst AJ, Castelle CJ, Butterfield CN, Hernsdorf AW, Amano Y, Ise K, Suzuki Y, Dudek N, Relman DA, Finstad KM, Amundson R, Thomas BC, Banfield JF 2016 A new view of the tree of life. Nat Microbiol 1:16048. doi:10.1038/nmicrobiol.2016.48. 27572647

[31] Feinstein LM, Sul WJ, Blackwood CB 2009 Assessment of bias associated with incomplete extraction of microbial DNA from soil. Appl Environ Microbiol 75:5428–5433. doi:10.1128/AEM.00120-09. 19561189 PMC2725469

[32] Klindworth A, Pruesse E, Schweer T, Peplies J, Quast C, Horn M, Glöckner FO 2013 Evaluation of general 16S ribosomal RNA gene PCR primers for classical and next-generation sequencing-based diversity studies. Nucleic Acids Res 41:e1. doi:10.1093/nar/gks808. 22933715 PMC3592464

[33] Shoemaker WR, Locey KJ, Lennon JT 2017 A macroecological theory of microbial biodiversity. Nat Ecol Evol 1:107. doi:10.1038/s41559-017-0107. 28812691

[34] R Core Development Team 2009 R: a language and environment for statistical computing, reference index version 2.8.1. R Foundation for Statistical Computing, Vienna, Austria.

[35] Hamilton SK, Doll JE, Robertson GP 2015 The ecology of agricultural landscapes: long-term research on the path to sustainability. Oxford University Press, New York, NY.

[36] Dell’Anno A, Stefano B, Danovaro R 2002 Quantification, base composition, and fate of extracellular DNA in marine sediments. Limnol Oceanogr 47:899–905. doi:10.4319/lo.2002.47.3.0899.

[37] Villarreal JV, Jungfer C, Obst U, Schwartz T 2013 DNase I and proteinase K eliminate DNA from injured or dead bacteria but not from living bacteria in microbial reference systems and natural drinking water biofilms for subsequent molecular biology analyses. J Microbiol Methods 94:161–169. doi:10.1016/j.mimet.2013.06.009. 23811209

[38] Lau JA, Lennon JT 2011 Evolutionary ecology of plant-microbe interactions: soil microbial structure alters selection on plant traits. New Phytol 192:215–224. doi:10.1111/j.1469-8137.2011.03790.x. 21658184

[39] Fierer N, Jackson JA, Vilgalys R, Jackson RB 2005 Assessment of soil microbial community structure by use of taxon-specific quantitative PCR assays. Appl Environ Microbiol 71:4117–4120. doi:10.1128/AEM.71.7.4117-4120.2005. 16000830 PMC1169028

[40] Sambrook J, Russell DW 2001 Molecular cloning: a laboratory manual. Cold Spring Harbor Laboratory Press, Cold Spring Harbor, NY.

[41] Caporaso JG, Lauber CL, Walters WA, Berg-Lyons D, Huntley J, Fierer N, Owens SM, Betley J, Fraser L, Bauer M, Gormley N, Gilbert JA, Smith G, Knight R 2012 Ultra-high-throughput microbial community analysis on the Illumina HiSeq and MiSeq platforms. ISME J 6:1621–1624. doi:10.1038/ismej.2012.8. 22402401 PMC3400413

[42] Needleman SB, Wunsch CD 1970 A general method applicable to the search for similarities in the amino acid sequence of two proteins. J Mol Biol 48:443–453. doi:10.1016/0022-2836(70)90057-4. 5420325

[43] Edgar RC, Haas BJ, Clemente JC, Quince C, Knight R 2011 UCHIME improves sensitivity and speed of chimera detection. Bioinformatics 27:2194–2200. doi:10.1093/bioinformatics/btr381. 21700674 PMC3150044

[44] Price MN, Dehal PS, Arkin AP 2010 FastTree 2: approximately maximum-likelihood trees for large alignments. PLoS One 5:e9490. doi:10.1371/journal.pone.0009490. 20224823 PMC2835736

[45] Lozupone CA, Knight R 2008 Species divergence and the measurement of microbial diversity. FEMS Microbiol Rev 32:557–578. doi:10.1111/j.1574-6976.2008.00111.x. 18435746 PMC2443784

[46] Schloss PD, Westcott SL, Ryabin T, Hall JR, Hartmann M, Hollister EB, Lesniewski RA, Oakley BB, Parks DH, Robinson CJ, Sahl JW, Stres B, Thallinger GG, Van Horn DJ, Weber CF 2009 Introducing Mothur: open-source, platform-independent, community-supported software for describing and comparing microbial communities. Appl Environ Microbiol 75:7537–7541. doi:10.1128/AEM.01541-09. 19801464 PMC2786419

[47] Muscarella ME, Jones SE, Lennon JT 2016 Species sorting along a subsidy gradient alters community stability. Ecology 97:2034–2043. doi:10.1890/15-2026.1. 27859189

[48] Smith B, Wilson JB 1996 A consumer’s guide to evenness indices. Oikos 76:70–82. doi:10.2307/3545749.

[49] Faith DP 1992 Conservation evaluation and phylogenetic diversity. Biol Conserv 61:1–10. doi:10.1016/0006-3207(92)91201-3.

