# Supplementary material for: How, When, and Where Relic DNA Affects Microbial Diversity
Source: mBio. 2018 Jun 19;9(3):e00637-18. doi: 10.1128/mBio.00637-18 (PMC6016248; doi:10.1128/mBio.00637-18)

Fig. S1. Additional detail on sampling-based and process-based models. Dissimilarity in composition of intact and total (relic + intact) communities from sampling-based simulation models. We populated relic communities from a lognormal species abundance distribution (SAD) with different degrees of evenness (E).

We calculated the parameters needed to obtain relic DNA proportions between 0.05 and 0.95 given the differential equations for our system:

$$\frac{dI}{dt} = rI - mI, \qquad \frac{dR}{dt} = mI - dR$$

where *I* is the number of sequences in the intact DNA pool, *R* is the number of sequences in relic DNA pool, *r* is the per capita birth rate of populations in *I*, *m* is the per capita mortality rate of bacterial populations in *I*, and *d* is the per capita degradation rate of sequences in *R*. The intact community is at steady state when $r = m$, and the relic community is at steady state when:

$$R = \frac{m \cdot I}{d}$$

Since the proportion of relic DNA (p) is calculated as:

$$p = \frac{R}{I + R}$$

we can substitute *R*:

$$p = \frac{\frac{mI}{d}}{I+\frac{mI}{d}},$$

which simplifies to:

$$p = \frac{\frac{m}{d}}{1+\frac{m}{d}}$$

Finally, we can solve for *d*:

$$d = \frac{m}{p} - m$$

Therefore, if given the mortality rate (m), we can calculate the degradation rate (d) needed to obtain any target proportion relic DNA (p).

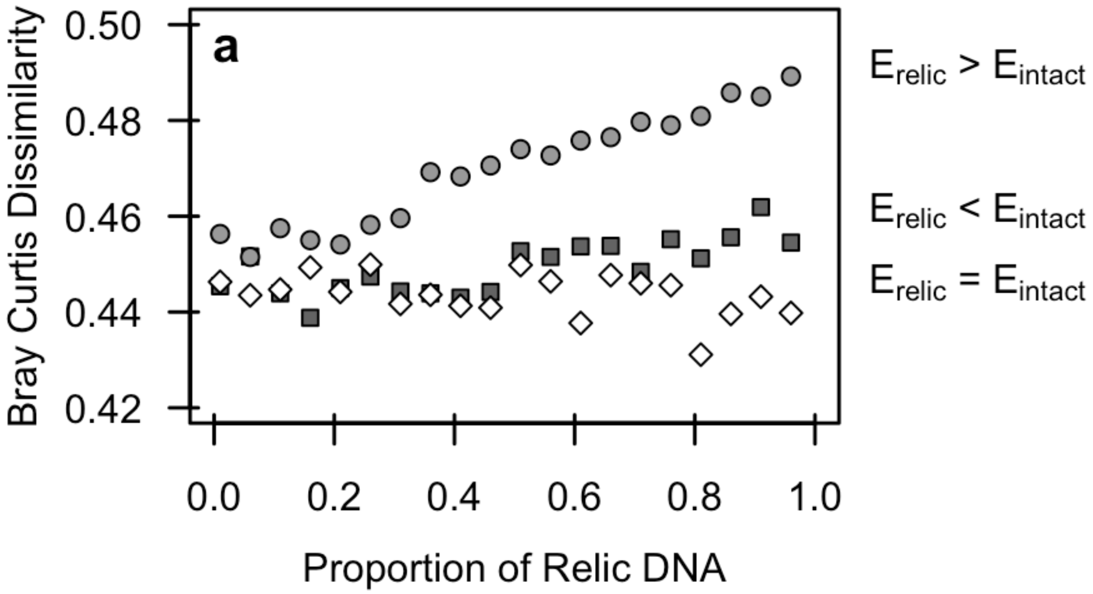

Supplement: FIG S1 [file mbo003183932sf1.pdf]
