# Supplementary material for: How, When, and Where Relic DNA Affects Microbial Diversity
Source: mBio. 2018 Jun 19;9(3):e00637-18. doi: 10.1128/mBio.00637-18 (PMC6016248; doi:10.1128/mBio.00637-18)
Supplement: TABLE S1 [file mbo003183932st1.pdf]

Table S1. Proportion of relic DNA in samples from different ecosystem types. The mean coefficient of variation (standard deviation / mean) for the16S rRNA qPCR assay applied to independent samples within a site was 0.24.

Sample #	Sample Name	Ecosystem type	Relic DNA
23	Feces1	gut	0.329
24	Feces2	gut	0.467
30	FecesDog	gut	0.401
31	FecesCow1	gut	0.617
32	FecesCat	gut	0.000
33	FecesCow2	gut	0.199
34	FecesHorse	gut	0.684
35	FecesRabbit	gut	0.170
2	KelloggDrain	sediment	0.503
7	Gull	sediment	0.648
9	Wintergreen	sediment	0.285
11	Austin	sediment	0.399
13	Woods	sediment	0.547
15	Crooked	sediment	0.062
16	Sherman	sediment	0.031
18	Duck	sediment	0.000
5	DF	soil	0.276
21	Kzoo	soil	0.167
22	Holland	soil	0.162
25	T7	soil	0.000
26	Entisol	soil	0.196
27	Spodosol1	soil	0.223
27	Spodosol2	soil	0.000
29	Histosol	soil	0.379
1	Gull	water	0.362
3	Augusta	water	0.578
4	KelloggDrain	water	0.363
8	Wintergreen	water	0.340
10	Austin	water	0.325
12	Woods	water	0.195
14	Crooked	water	0.454
17	Duck	water	0.290
19	Sherman	water	0.590
20	Sherman	water	0.830
