# Supplementary figures and images for: How, When, and Where Relic DNA Affects Microbial Diversity
Source: mBio. 2018 Jun 19;9(3):e00637-18. doi: 10.1128/mBio.00637-18 (PMC6016248; doi:10.1128/mBio.00637-18)

**Fig. S2.** Flow diagram depicting how a sample was processed to quantify the proportion of relic DNA.

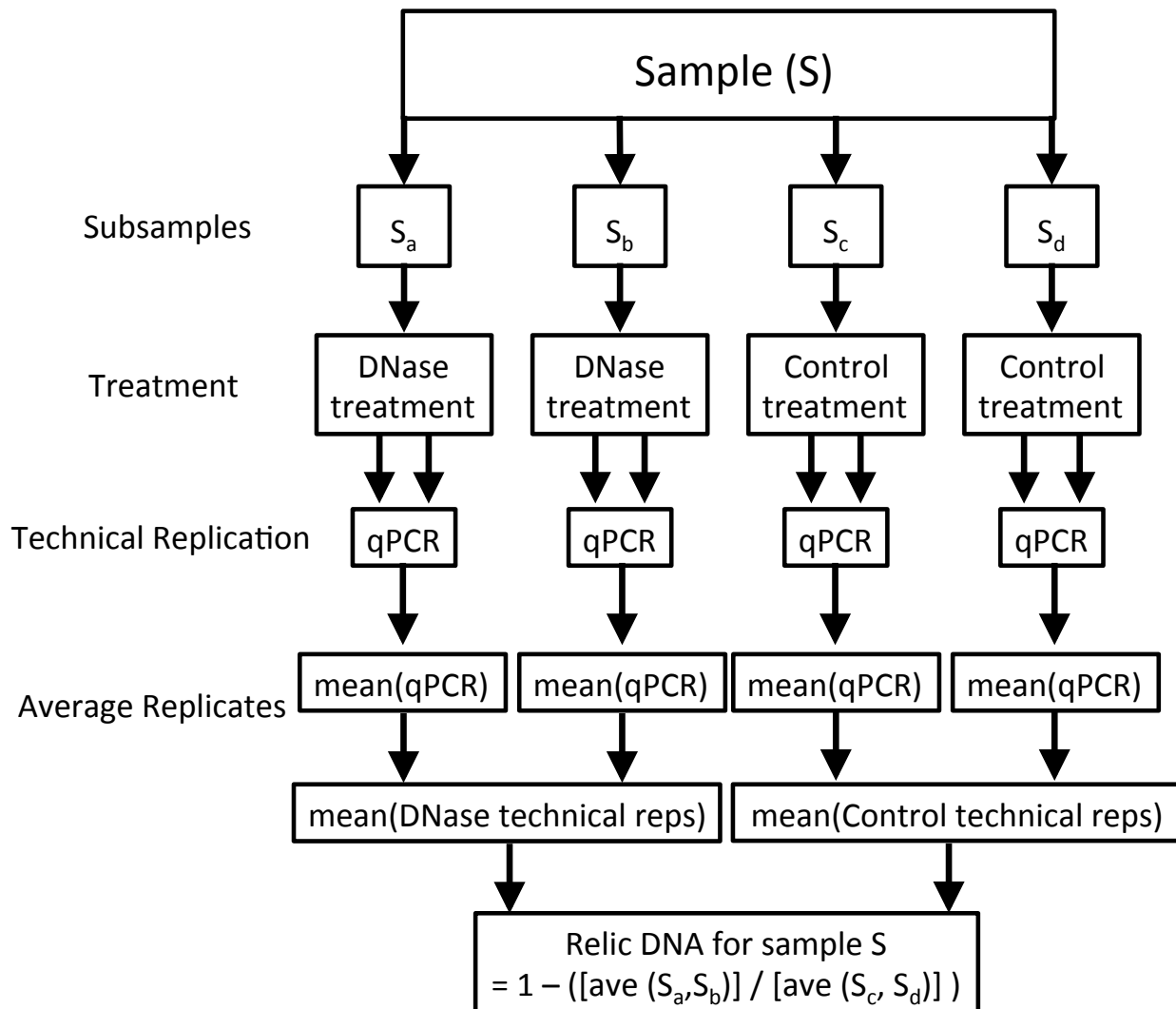

Supplement: FIG S2 [file mbo003183932sf2.pdf]
