# Supplementary material for: How, When, and Where Relic DNA Affects Microbial Diversity
Source: mBio. 2018 Jun 19;9(3):e00637-18. doi: 10.1128/mBio.00637-18 (PMC6016248; doi:10.1128/mBio.00637-18)
Supplement: FIG S3 [file mbo003183932sf3.pdf]

Fig. S3. Effect of DNase I concentration on the estimated proportion of relic DNA in soil. We conducted an experiment to estimate the minimum concentration of DNase needed to remove ambient amounts of relic DNA from an environmental sample. The experiment was conducted using a soil sample collected from a Deciduous Forest (DF) plot at the W.K. Kellogg Biological Station (KBS) Long-Term Ecological Research (LTER) site. We altered the amount of DNase I in the master mix (2, 5, 10, 20, 40, 60 μL), which corresponded to DNase I concentrations of 0.04, 0.10, 0.20, 0.40, 0.80, 1.20 U/μL. We then measured the amount of relic DNA in each of the soil subsamples using quantitative PCR as described in the Methods section of the main text. We used a Michaelis-Menten function to describe the effect of enzyme concentration on the estimated proportion of relic DNA in the sample: Relic DNA = ($Relic\ DNA_{max}$ * *DNase*) / (K_{DNase} + *DNase*), where $Relic\ DNA_{max}$ is the maximum proportion of relic DNA measured, *DNase* is the final concentration of DNase I in the enzyme incubation, and K_{DNase} is the concentration of DNase equal to one-half $Relic\ DNA_{max}$. We fit the data to the Michaelis-Menten function using maximum likelihood procedures implemented with the `bbmle` package in R. Based on our analysis, we obtained the following parameter values: Relic DNA_{max} = 0.58 ± 0.033 and K_{DNase} = 0.06 ± 0.016, which corroborates our efficiency findings and suggests our estimates of relic DNA are robust for ranges of DNA concentrations between 0.20 and 1.20 U/μL, even in a soil sample where protection mechanisms could potentially interfere with the activity of nucleases.

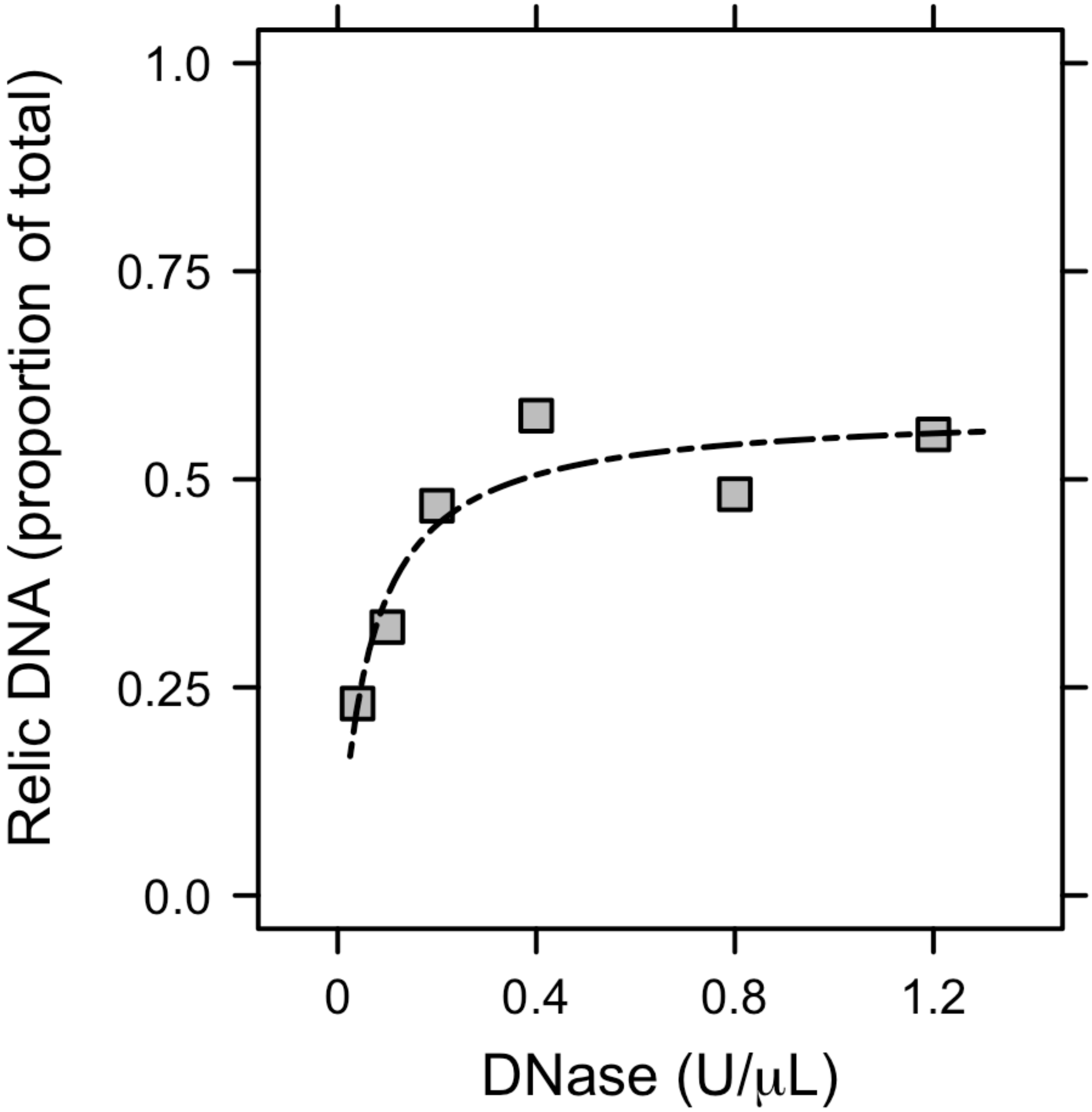

Relic DNA (proportion of total)
1.0
0.75
0.5
0.25
0.0
0
0.4
0.8
1.2
DNase (U/μL)
