# Supplementary material for: How, When, and Where Relic DNA Affects Microbial Diversity
Source: mBio. 2018 Jun 19;9(3):e00637-18. doi: 10.1128/mBio.00637-18 (PMC6016248; doi:10.1128/mBio.00637-18)
Supplement: FIG S4 [file mbo003183932sf4.pdf]

Fig. S4. Efficiency of quantifying relic DNA. We conducted an experiment to estimate the efficiency of removing relic DNA based on the DNase treatment described in the Methods section of the main text. We collected a soil core (0 - 10 cm) from Dunn Woods on the Indiana University campus in August 2017. In the laboratory, 0.1 g of the homogenized soil sample was diluted into 5 mL of autoclaved nanopure water. We pipetted 500 μL of the soil solution into 2 mL centrifuge tubes and then spiked in 1 μL of a 19 ng/μL stock of a 16S rRNA amplicon from a bacterial isolate. In triplicate, we then quantified 16S rRNA gene copy abundance in unspiked subsamples, spiked subsamples without DNase, and spiked subsamples treated with DNase following procedures outlined in the Methods section of the main text. With these data, we quantified the efficiency of DNase treatment as 1 - (DNA_{post} / DNA_{add}), where DNA_{add} is the gene copy abundance in the sample following the 16S rRNA spike and DNA_{post} is the gene copy abundance in the spiked sample following DNase treatment. We estimated that DNase treatment removed 98 % ± 1.10 % of the dissolved DNA that was spiked into the soil sample.

We also estimated the efficiency of relic DNA quantification using the propidium monoazide (PMA) kit (Carini et al. 2016). When PMA binds with free DNA, it prevents amplification in qPCR assays. Therefore, PMA-untreated samples represent total DNA (relic DNA + intact DNA), while PMA-treated samples should only amplify DNA contained in intact cells. With the exception of not pelleting cells prior to DNA extraction, we followed the manufacturer's (Biotium) recommended procedures in combination with the procedures described in the Methods section of the main text for qPCR. We estimated the PMA treatment prevented amplification of 70 % ± 12.4 % of the dissolved DNA that was spiked into the soil sample. A two-tailed *t*-test assuming unequal variances revealed that the efficiency of the two methods was comparable ($t_{3.15}$ = -2.19, P = 0.115).

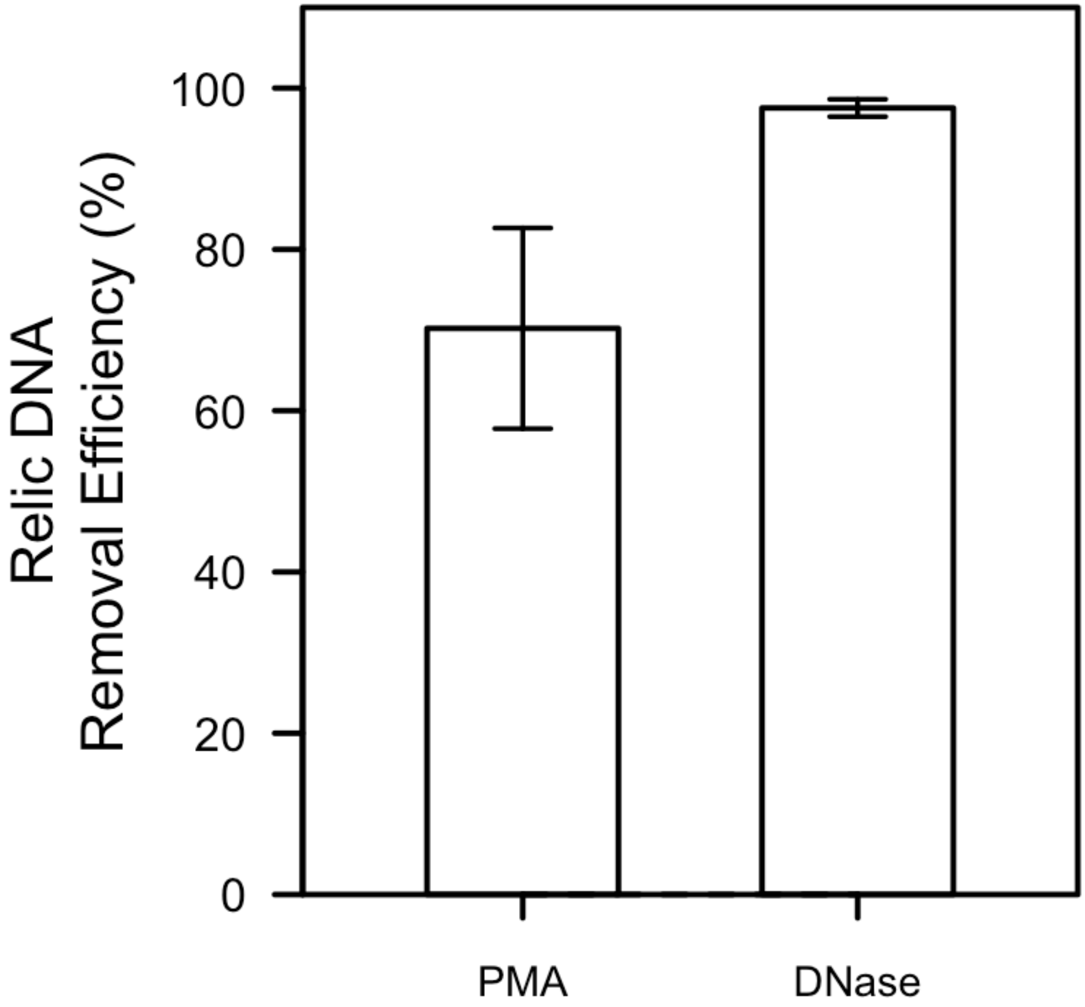

Relic DNA
Removal Efficiency (%)
100
80
60
40
20
0
PMA
DNase
