# Supplementary material for: How, When, and Where Relic DNA Affects Microbial Diversity
Source: mBio. 2018 Jun 19;9(3):e00637-18. doi: 10.1128/mBio.00637-18 (PMC6016248; doi:10.1128/mBio.00637-18)

Fig. S5. Principal Coordinates Analysis (PCoA) on log_{10}-transformed relative abundances using **a** Bray-Curtis and **b** UniFrac distance metrics to test for the effects of relic DNA removal on community composition. "DNase" symbols refer to samples where relic DNA was removed by DNase treatment (a.k.a., intact DNA); "Control" symbols refer to samples that were not treated with DNase and thus contain intact + relic DNA (a.k.a., total DNA).

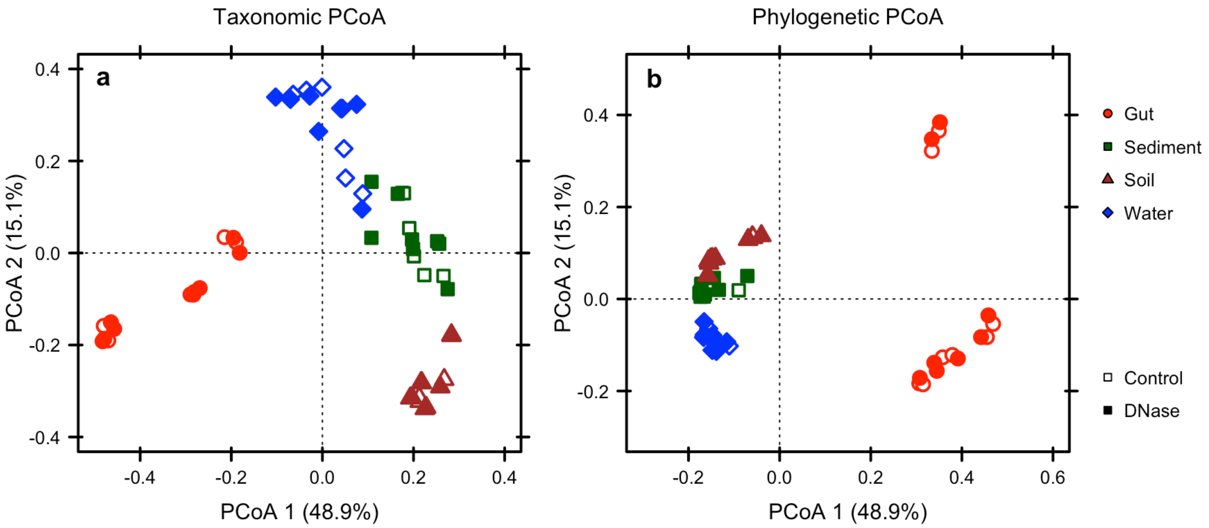

Supplement: FIG S5 [file mbo003183932sf5.pdf]
