# Supplementary material for: How, When, and Where Relic DNA Affects Microbial Diversity
Source: mBio. 2018 Jun 19;9(3):e00637-18. doi: 10.1128/mBio.00637-18 (PMC6016248; doi:10.1128/mBio.00637-18)

Fig. S6. Centroid distance ratios used to quantify effects of relic DNA on beta diversity. First, we calculated the mean centroid distance for bacterial composition from the total (i.e., intact + relic) DNA pool for each of the four ecosystem types. Distances were calculated in all dimensions of the PCoA, but for illustrative purposes we only show this procedure for the first (x) and second (y) axes of the taxonomic data (Bray-Curtis distance). Ratios were then calculated on a per-sample basis as the distance for the total subsample divided by the distance for the intact subsample. Red solid line = total DNA; Blue dashed line = intact DNA.

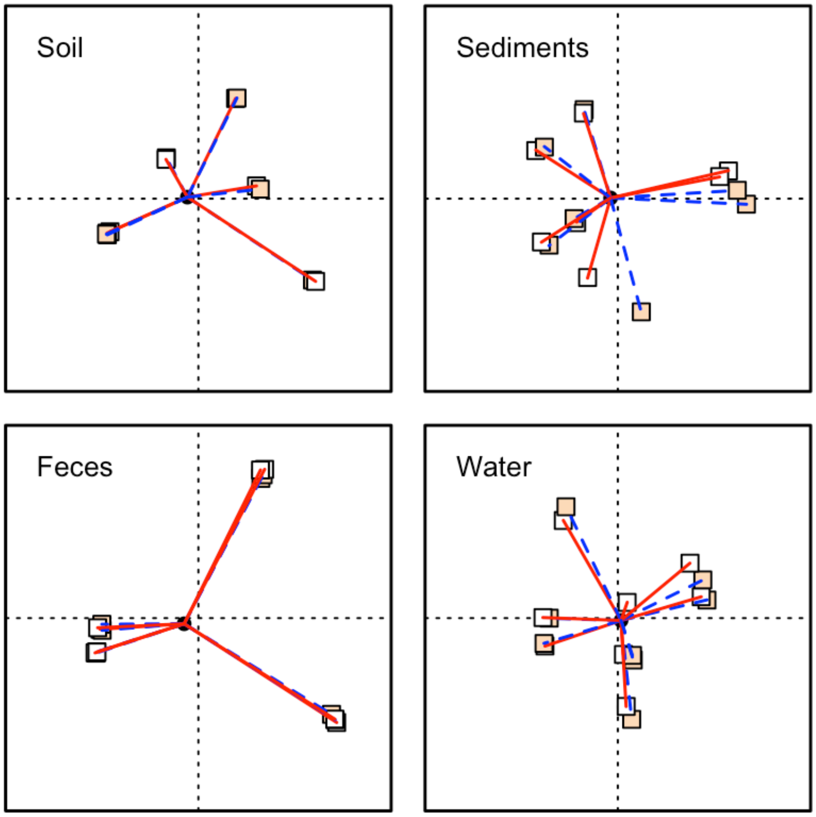

Supplement: FIG S6 [file mbo003183932sf6.pdf]
