# Supplementary material for: How, When, and Where Relic DNA Affects Microbial Diversity
Source: mBio. 2018 Jun 19;9(3):e00637-18. doi: 10.1128/mBio.00637-18 (PMC6016248; doi:10.1128/mBio.00637-18)
Supplement: TABLE S2 [file mbo003183932st2.pdf]

Table S2. Estimates of diversity within samples from different ecosystem types. Richness was calculated as the number of operational taxonomic units (97% sequence similarity of the 16S rRNA gene), evenness was calculated using Simpson's evenness index, and phylogenetic diversity was calculated using Faith's *D* index. The intact DNA samples were treated with DNase to remove relic DNA. The total samples were not treated with DNase and thus contained intact and relic DNA. Values are means and standard error of the means (in parentheses).

**Ecosystem**	**Richness**		**Evenness**		**Faith's *D***	
	Intact	Total	Intact	Total	Intact	Total
Gut	1435	1474	0.379	0.375	167	168
	(402.7)	(463.2)	(0.0172)	(0.1481)	(41.8)	(44.2)
Soil	2950	3084	0.240	0.240	398	415
	(387.6)	(397.8)	(0.0568)	(0.0587)	(46.1)	(48.6)
Lake	1362	1344	0.151	0.147	295	297
	(359.8)	(235.6)	(0.0280)	(0.0268)	(58.0)	(39.1)
Sediments	3875	4432	0.199	0.187	581	657
	(517.0)	(345.6)	(0.0251)	(0.0227)	(57.5)	(33.7)
