# Supplementary material for: How, When, and Where Relic DNA Affects Microbial Diversity
Source: mBio. 2018 Jun 19;9(3):e00637-18. doi: 10.1128/mBio.00637-18 (PMC6016248; doi:10.1128/mBio.00637-18)
Supplement: FIG S7 [file mbo003183932sf7.pdf]

Fig. S7. Results from sampling-based model simulating conditions hypothesized to affect bias in estimates of microbial diversity. We express standardized estimates of diversity using richness ratios, which is the quotient of taxon richness in the total and intact DNA pools. As described in the main text, with increasing relic DNA pool size, physical protection leads to an underestimation of taxon richness while hot spots of activity lead to the underestimation of taxon richness. We also evaluated how immigration of relic DNA from a regional species pool affects estimates of microbial diversity. Because the regional species pool is more diverse than the local community, an increased immigration rate into the relic DNA pool decouples the dynamics between the intact and relic DNA pools leading to elevated bias.

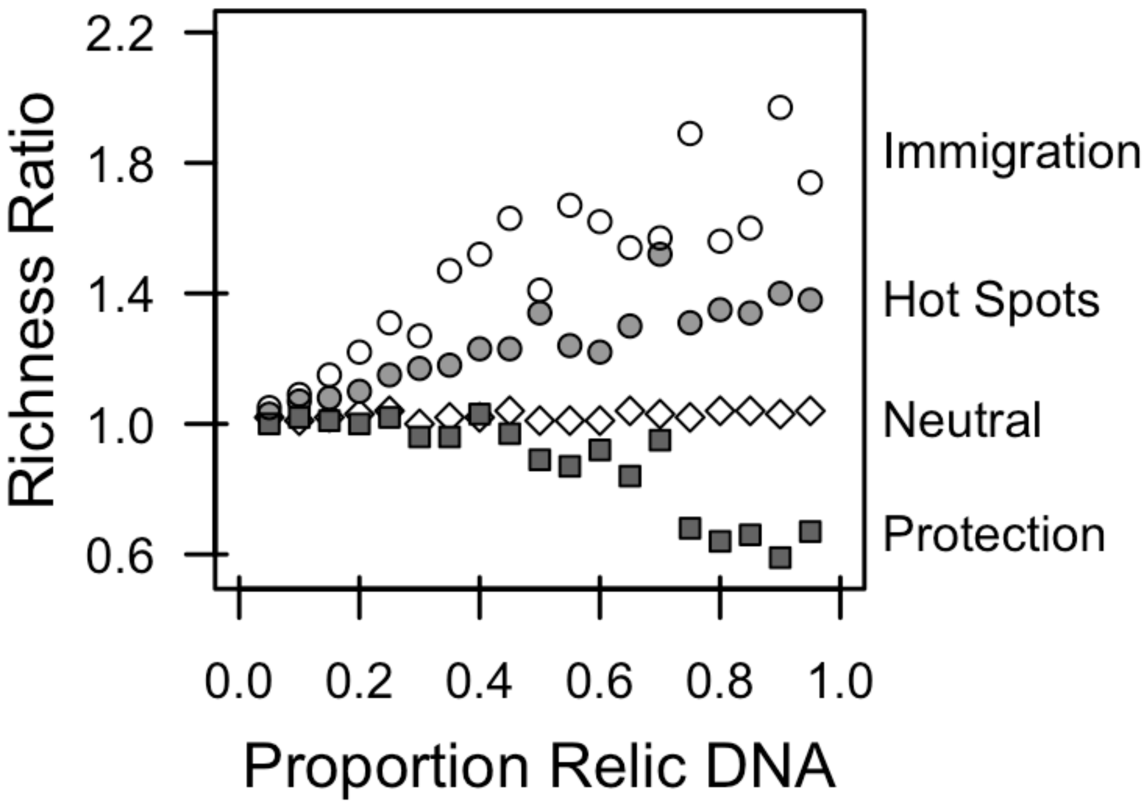
Richness Ratio
2.2
1.8
1.4
1.0
0.6
0.0
0.2
0.4
0.6
0.8
1.0
Proportion Relic DNA
Immigration
Hot Spots
Neutral
Protection
